# The Promising Potency of Sodium–Glucose Cotransporter 2 Inhibitors in the Prevention of and as Treatment for Cognitive Impairment Among Type 2 Diabetes Patients

**DOI:** 10.3390/biomedicines12122783

**Published:** 2024-12-06

**Authors:** Yibin Zhang, Xiaobin Liao, Jialu Xu, Jiaxin Yin, Shan Li, Mengni Li, Xiaoli Shi, Shujun Zhang, Chunyu Li, Weijie Xu, Xuefeng Yu, Yan Yang

**Affiliations:** 1 Department of Endocrinology, Tongji Hospital, Tongji Medical College, Huazhong University of Science and Technology, Wuhan 430030, China; 17343975167@163.com (Y.Z.); 19977295795@163.com (X.L.); jlxu93@163.com (J.X.); yinjiaxin6892@163.com (J.Y.); lishandoctor@163.com (S.L.); tj_lmn@163.com (M.L.); fll766@163.com (X.S.); sjzhang0407@163.com (S.Z.); chunyu_tongji@foxmail.com (C.L.); xwj.07@163.com (W.X.); xfyu188@163.com (X.Y.); 2 Second Clinical College, Tongji Medical College, Huazhong University of Science and Technology, Wuhan 430030, China; 3 Branch of National Clinical Research Center for Metabolic Diseases, Wuhan 430030, China

**Keywords:** type 2 diabetes mellitus (T2DM), cognitive impairment (CI), sodium–glucose cotransporter 2 inhibitors (SGLT2i), mechanisms and pathways, clinical evidence

## Abstract

Type 2 diabetes mellitus (T2DM), accounting for the majority of diabetes mellitus prevalence, is associated with an increased risk of cognition decline and deterioration of cognition function in diabetic patients. The sodium–glucose cotransporter 2 (SGLT2), located in the renal proximal tubule, plays a role in urine glucose reabsorption. SGLT2 inhibitors (SGLT2i), have shown potential benefits beyond cardiac and renal improvement in preventing and treating cognitive impairment (CI), including mild cognitive impairment, Alzheimer’s disease and vascular dementia in T2DM patients. Studies suggest that SGLT2i may ameliorate diabetic CI through metabolism pathways, inflammation, oxidative stress, neurotrophic factors and AChE inhibition. Clinical trials and meta-analyses have reported significant and insignificant results. Given their vascular effects, SGLT2i may offer unique protection against vascular CI. This review compiles mechanisms and clinical evidence, emphasizing the need for future analysis, evaluation, trials and meta-analyses to verify and recommend optimal SGLT2i selection and dosage for specific patients.

## 1. Introduction

DM is a metabolic syndrome characterized by chronic hyperglycemia. T2DM, as a subtype of DM, originates from insulin resistance, leading to progressive pancreatic β cell failure and insulin insufficiency [1,2,3]. It is estimated that 529 million people worldwide live with diabetes, with a global age-standardized total diabetes prevalence of 6.1%, and this number is projected to exceed 1.31 billion by 2050 [4]. T2DM accounts for nearly 96.0% of all diabetes cases [4]. Additionally, the number of people with IGT and IFG is expected to rise from 464 million and 298 million in 2021 to 638 million and 414 million, respectively, by 2050, posing a huge burden on global health [5].

CI, which is classified into MCI and dementia, is defined as a significant and progressive decline in one or more cognitive domains compared to a person’s previous level, including learning and memory, social cognition, complex attention, executive function, perceptual-motor function and language [6]. MCI, also known as predementia, is distinguished from dementia by its impact on functional abilities conducting one’s daily life, with no insignificant impairment in social or occupational functioning [6]. Dementia is a group of heterogeneous diseases with various etiologies, including AD, VaD and other subtypes [7]. AD, the most common subtype of dementia, typically presents as an amnestic variant [8]. Confirming a diagnosis of AD requires neuropathologic evidence of both amyloid plaques from Aβ peptides and NFTs composed of hyperphosphorylated and abnormally folded tau proteins [9]. VaD, the second most common cause of dementia, accounts for 15% of cases [10] and is characterized by vascular changes, such as infarcts, atherosclerosis, arteriolosclerosis and CAA, which have independent and additive effects on the risk of CI [11]

Diabetic patients are at significantly higher risk of various complications, including CI. A population-based prospective follow-up study conducted by A. Ott et al. in 1996 suggested that diabetes may increase the risk of dementia onset, with VaD contributing modestly to the incidence of dementia in T2DM [12]. Recent analysis of cohort studies has identified diabetes as a risk factor for dementia, with a weighted population attributable fraction of 1.9% [13]. Another meta-analysis of DM patients in Africa showed that poorly controlled diabetes and a diabetes duration of over 10 years are risk factors associated with CI, with pooled OR (a statistical measure used to summarize the OR from multiple studies in a meta-analysis) of 5.85 and 1.13, respectively [14]. In 2024, a systematic umbrella review of diabetic CI evidence revealed a significant association between diabetes and an increased risk of AD and VaD [15]. A longitudinal study in 2024 reported that patients experienced a significantly faster rate of cognitive decline after the onset of diabetes (difference in slope after diabetes onset −0.23, 95%CI −0.043 to −0.004, *p* = 0.019), concluding that diabetes might accelerate the deterioration of cognitive function [16]. The ADA in 2024 stated that elderly individuals with diabetes are at higher risk of CI and recommended they be screened and monitored for CI using simple assessment methods such as the MMSE or MoCA [17].

SGLT2 is primarily expressed in the renal proximal tubules and is responsible for glucose reabsorption. SGLT2i, including canagliflozin, dapagliflozin, empagliflozin, ertugliflozin and sotagliflozin, act as the antagonists of SGLT2, leading to reduced glucose reabsorption and glycosuria, and have been shown to benefit metabolism and cardiorenal outcomes in patients with or without diabetes [18]. Meta-analyses have reported more compelling results. In 2024, a Bayesian network meta-analysis favored metformin and SGLT2i to reduce the risk of dementia and AD compared to DPP−4i, SUs and TZDs [19]. A network meta-analysis in 2023 concluded the treatment effects of SGLT2i were comparable to GLP-1 Ras and superior to TZDs and DPP-4i for the cognition outcomes related to T2DM, as measured by SUCRA [20]. Antidiabetic agents are increasingly recognized for their potential in treating T2DM-associated CI.

To elucidate the mechanism of SGLT2i in treating CI related to diabetes and T2DM, this review aims to summarize the pathogenesis and treatments and compile experiments and reviews to provide a comprehensive understanding after secondary integration. In addition to AD, VaD is also discussed. Clinical trials and evidence-based meta-analyses are sorted to support and verify the use of SGLT2i in this field. Since evidence on VaD is relatively scarce, the association between SGLT2i and ACVEs, such as stroke, ischemic and hemorrhage cerebral diseases, has been included based on the pathophysiology of the VaD.

## 2. Potential Pathophysiological Pathways About Diabetic CI

(A) Impaired metabolism through the insulin pathway and others, (B) inflammation and oxidative stress and (C) cerebral vessel pathology in diabetes might all contribute to CI, its subtypes, or mixed types. Many experiments focus on AD, but VaD in diabetes should not be overlooked. Future studies on vascular factors contributing to CI are warranted.

### 2.1. GSK3β: Impaired Insulin Signal Pathways for Insulin Resistance

Intracellularly, insulin binds to IR and regulates glycogen synthesis via the IRS/PI3K/AKT/GSK3β pathway, where GSK3β negatively affects glucose tolerance and homeostasis is inhibited by AKT in this signaling pathway [21,22]. Similarly, insulin signaling in the brain consists of IR/IRS/PI3K/AKT regulating food intake and endogenous glucose production, etc., based on animal studies and intranasal insulin studies [23]. When insulin resistance occurs in the brain, a reduced amount of insulin transported by the BBB causes a relative insulin deficiency and the inability of the insulin signaling pathway, which ultimately impairs central and peripheral metabolic function, possibly affecting mood and cognition [23]. The leptin receptor in the CNS regulates appetite, body weight, neuroendocrine functions and glycemia, initiating the cascade of PI3K/AKT [24]. T2DM patients with obesity commonly experience leptin resistance, and there is evidence of the therapeutic potential of leptin due to its glucoregulatory and antidiabetic actions based on clinical trial results [24]. When the Wnt/β–catenin pathway is disheveled, leptin is activated by the Wnt-coreceptor low-density lipoprotein receptor-related protein complex and also acts as an inhibitor of GSK3β [25]. Experiments have shown evidence that attributes tau hyperphosphorylation, an AD biomarker, to PI3K/AKT/GSK3β signaling pathway damage due to diabetes mellitus. As a kinase, the derepressed GSK3β increases phosphorylation of the tau protein in the damaged signaling pathway, ultimately contributing to CI in AD or poststroke dementia, etc. [26,27,28,29,30,31,32,33,34,35,36,37,38,39,40,41,42,43,44,45,46,47,48]. Leptin produced by high-intensity interval training ameliorates tau phosphorylation via PI3K/AKT/GSK3β downstream to bypass leptin signaling suffering caused by leptin receptor insufficiency in the hippocampus due to T2DM [49]. Canonical Wnt/β-catenin repairs the insulin/PI3K/AKT/GSK3β function, showing the neuroprotective potential of manually amplifying the Wnt/β-catenin/Ins2 pathway [50,51,52,53,54]. During T2DM, elevated glucocorticoids and hyperglycemia might directly affect GSK3β, accelerating the progression of tau pathology or AD [55,56]. All experiments have demonstrated that GSK3β and relevant pathways might be therapeutic targets in the future.

### 2.2. IDE: Insulin Resistance and Aβ Protein

In addition to the tau protein, another biomarker of AD is the Aβ protein, whose deposition is involved in core pathophysiological mechanisms [9,57]. The generation of APP requires cleavage by the β-site Aβ precursor protein-cleaving enzyme 1 [58]. When experiencing impaired insulin-related signaling pathways, insulin resistance and hyperinsulinemia, IDE, a degrading enzyme of insulin and Aβ, is considered as a bridge linking diabetes and CI and AD via the potential signaling pathways of PI3K/AKT/GSK3β, PPARγ and NFκB [59]. With diverse cellular localization, the IDE becomes disabled due to the aberrant S-nitrosylation and competitive blocking by insulin at the Aβ binding site, which might provide a rough explanation of how the IDE connects AD with T2DM with a background of hyperglycemia and hyperinsulinemia caused by insulin resistance [60,61,62,63,64,65]. Experiments have demonstrated that the expression, transcription, translation and activity of the cerebral IDE affected by age, insulin or glycemia participate in the onset and progression of CI and AD [66,67,68,69,70,71,72,73,74,75,76,77,78,79,80,81,82,83,84,85,86]. As mentioned above, CAA is one of the key vascular wall modifications contributing to VaD [11]. It should be recognized that not only does the IDE promote the progress of the AD stage but also VaD in T2DM, meaning that the IDE might contribute to VaD or mixed dementia [87,88,89]. On the contrary, the role of insulin is disputed, with some studies concluding a protective role, whereas cerebral hyperinsulinemia is considered a risk factor for CI due to T2DM, which probably contributes to the diabetic model or various other standpoints [90,91,92,93]. In another trial, the hypothesis that hyperinsulinemia would significantly elevate the Aβ load and thus exacerbate the extent of AD is challenged by the results showing no influence of the Aβ load on the brains of 134 diabetic and 567 non-diabetic subjects [94]. There are also studies reporting Aβ deposition where the IDE is not affected [90,95].

IDEs inactivated by an oxidant might be the dominant reason for the massive Aβ accumulation. Another explanation is that the state called hyperinsulinemia in the brain is relative and regional due to insulin resistance, where the insulin receptor becomes insensitive. Not only relative hyperinsulinemia but also oxidants, such as NO, attack the IDE, which ultimately loses its proteolysis function towards insulin and Aβ. With insulin accumulating regionally, this positive feedback deteriorates cerebral Aβ homeostasis, and then CI is classified as a branch of the pathophysiological mechanism of T2DM.

### 2.3. Neuroinflammation and Oxidative Stress

Microglial, the main defenders in the brain, can be activated by a range of insults, including hyperglycemia, metabolic stress, ischemia and hypoxia in diabetes, as well as Aβ proteins. It is then polarized into the proinflammatory M1 classification with upregulated inflammatory mediators such as IL-1β, IL-, and TNF-α [96]. In addition to inactivating the IDE, as mentioned above, oxidants might have a direct effect on the CNS in diabetes. Coupled with an inflammatory reaction, cerebral glycemia overload damages the balance between glycogenesis and glycolysis and mitochondria function, therefore producing excessive ROS, such as superoxide (O_2_^−^), hydroxyl (HO) and hydrogen peroxide (H_2_O_2_), via NFκB and NOX2, all of which ultimately results in DNA oxidation, lipid peroxidation, advanced oxidation of proteins, and the formation of AGEs [96]. In a combined analysis of the brain transcriptome from db/db mice with CI, 222 upregulated gene sets and 85 downregulated gene sets have been defined, mainly related to mitochondrial respiratory and oxidative stress, and glycolytic and inflammation [97]. Recently, basic studies involving complex pathway crosstalk have deeply explored the role of M1 microglial polarization, inflammatory signaling pathways and ROS, which injure neurons and cause apoptosis or necrosis, in the decline in cognition function related to diabetes [98,99,100,101,102,103,104,105,106]. In a 12-year followed-up cohort of older adults with healthy cognition function (*n* = 1840) or MCI (*n* = 682), diabetes patients (HbA1c ≥ 7.5%) had a significantly higher (triple) risk of the progression of MCI to dementia (HR 3.30, 95%CI 1.02–9.93) accompanied by elevated CRP compared to normoglycemia, while those with diabetes with normal CRP showed an insignificant risk (HR 0.9, 0.48–1.67) [107].

### 2.4. Cerebral Vascular Modification

In 2024, a systematic review and meta-analysis focusing on stroke sorted the evidence on risk factors for poststroke CI and dementia, reporting that diabetes is a significant treatable risk factor for VaD, independent of age and stroke severity [108]. According to the vascular impairment of cognition classification consensus study, the subtypes of VaD include poststroke dementia and mixed dementias, such as subcortical ischemic VaD and multiple-infarct dementia [109]. Another classification defined subtypes of VaD from another perspective: multi-infract (cortical VaD), small vessel (subcortical VaD), strategic infarct, hypoperfusion and hemorrhagic dementias; hereditary VaD and AD with cardiovascular disease [10]. Obviously, VaD is a potential secondary outcome of cerebrovascular disease.

When vascular risk factors strike cerebral vessels, vascular wall modification occurs, including atherosclerosis, arteriolosclerosis and CAA, followed by tissue injuries such as macroscopic infarcts, microinfarcts, hemorrhages, microbleeds and white matter hyperintensities, along with neurodegeneration, including neuronal and/or volume loss, oligodendrocyte loss, astrocytosis and microgliosis, elucidating the mechanism of VaD [11]. Diabetes itself confers an independent risk of atherosclerotic cardiovascular disease, such as coronary atherosclerotic heart disease and cerebral atherosclerosis [110]. The main risk factors associated with arteriolosclerosis are hypertension and diabetes, and T2DM is associated with an increased risk of brain arteriolosclerosis, small and large brain infarcts and white matter hyperintensity [111].

Therefore, when referring to the preventive and treatment effects of SGLT2i, the vascular effects have been summarized in response to the rare outcomes of VaD reported in clinical trials.

## 3. SGLT2i Improving CI Among Patients with T2DM

SGLT2, encoded by the *SLC5A2* gene, is responsible for glucose reabsorption primarily in the kidney. It is a new target for treating diabetes as it actively transports Na^+^ and glucose with a ratio of 1:1 across cell membranes as they are energized by the sodium gradient [112]. Almost all SGLT2 is located in the renal proximal tubule, performing 90% of glucose reabsorption, with the remaining part is performed by SGLT1 [113]. When it binds to an inhibitor, the activity of SGLT2 effectively decreases, resulting in reducing glucose reabsorption, promoting urinary glucose excretion, and exerting glucose-lowering effects, all of which ultimately relieve the sodium and glucose toxicity in the proximal tubule [113].

SGLT2i, including canagliflozin, dapagliflozin, empagliflozin, ertugliflozin, approved by the European Medicines Agency and the Federal Drug Administration, and sotagliflozin approved only by the European Medicines Agency for use in type 1 diabetes, as well as others still in trials, have been clinically observed to provide T2DM patients with extra cardiorenal benefits in the context of heart failure or CKD [18]. In summary, mechanisms underlying the cardiovascular and renal protective effects of SGLT2i include inhibiting myocardial Na^+^/H^+^ exchange, reducing cardiomyocyte apoptosis and improving myocardial fibrosis, reducing the synthesis of adipokines, cytokines and epicardial adipose tissue, improving glomerular hyperfiltration, reducing renal oxygen consumption and inflammatory reactions, etc. [113]. In addition to cardiorenal outcomes, it has been summarized recently that SGLT2i could improve HRQoL by decreasing the multimorbidity risk, lowering body weight, preventing depression incidence and improving physical function [114]. In the standards of diabetes care-2024 by the ADA, it is recommended that the treatment of adult T2DM patients with cardiovascular or/and renal risk should include SGLT2i and/or GLP-1 Ras [115]. However, even with such extra benefits when treating T2DM, there are possible adverse effects, such as polyuria and urinary tract infections, which might lead to treatment discontinuation [18].

Clinical trials on SGLT2i as preventive and treating agents have been collected to fully understand their impact. Meta-analyses synthesizing the results are also in this article. Here, an attempt is made to provide a comprehensive conclusion, with experiments and reviews summarized.

### 3.1. Evidence from Primary and Secondary Analysis of Clinical Trials

#### 3.1.1. Observational Clinical Trials and RCTs

**Figure 1:**
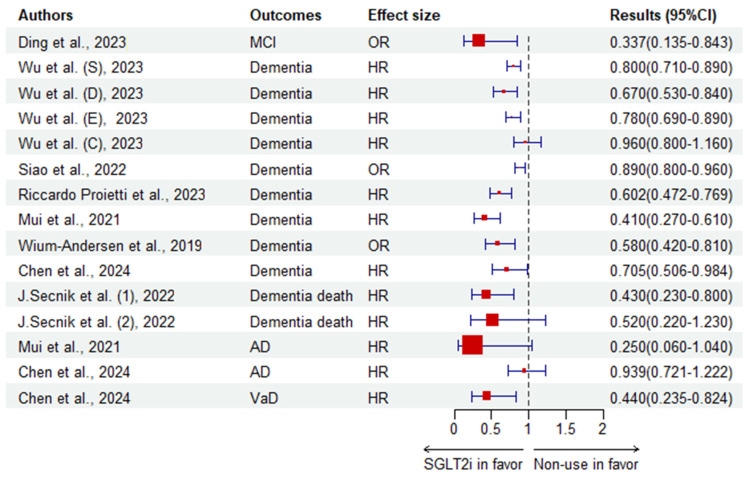
Results of clinical trials using binary variables to describe the effects of SGLT2i against CI compared to non-use. S, SGLT2i; Dapa, dapagliflozin; Empa, empagliflozin; Cana, canagliflozin; (1) where the population was diagnosed as having dementia at baseline; (2) where the population was free of dementia. The blue line represents the 95% confidence interval, which is insignificant when 7intersecting the vertical dotted line [116,118,119,121,122,125,126,127]. The red box indicates the trial result and standard error size of the result. Same for the following Figure 2, Figure 3, Figure 4, Figure 5, Figure 6 and Figure 7.

**Figure 2:**
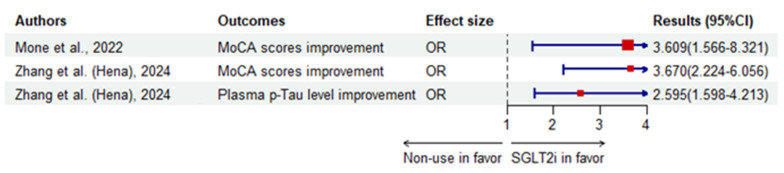
The improving effects of SGLT2i compared to non-use. Hena, henagliflozin [117,124].

**Figure 3:**
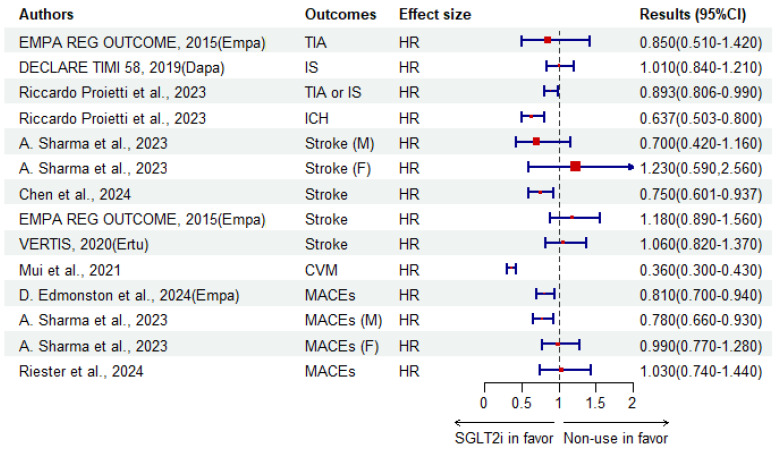
The association between SGLT2i and adverse cerebral vascular events compared to non-use. Empa, empagliflozin; Dapa, dapagliflozin; Ertu, ertugliflozin. (M) where the HR describes the risk among male patients; (F) where the HR describe the risk among female patients [121,125,126,128,129,130,131,132,133].

**Figure 4:**
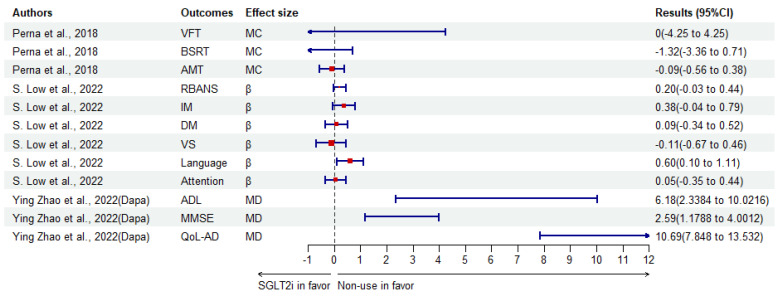
The results of continuous variables describing the protective effects of SGLT2i compared to non-use. Dapa, dapagliflozin; β, coefficient in multivariable regression analysis [123,134,135].

**Figure 5:**
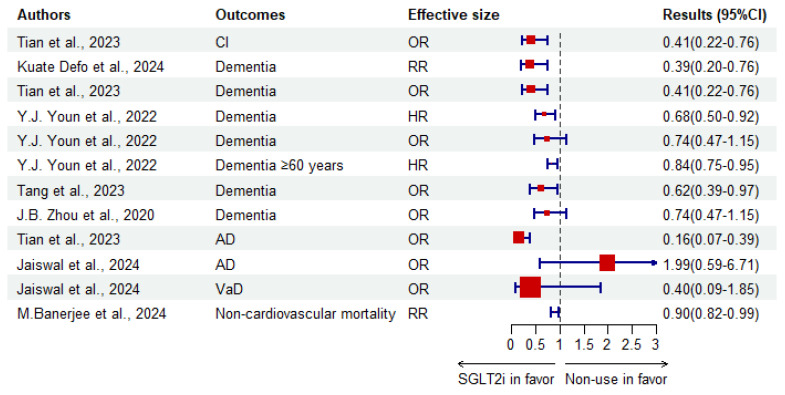
Forest plot of meta-analyses which pool the results of SGLT2i against CI in T2DM compared to non-use. Non-cardiovascular mortality includes infectious diseases, cancers, lung diseases, dementia, renal diseases and other external causes [15,20,136,137,138,139,140].

**Figure 6:**
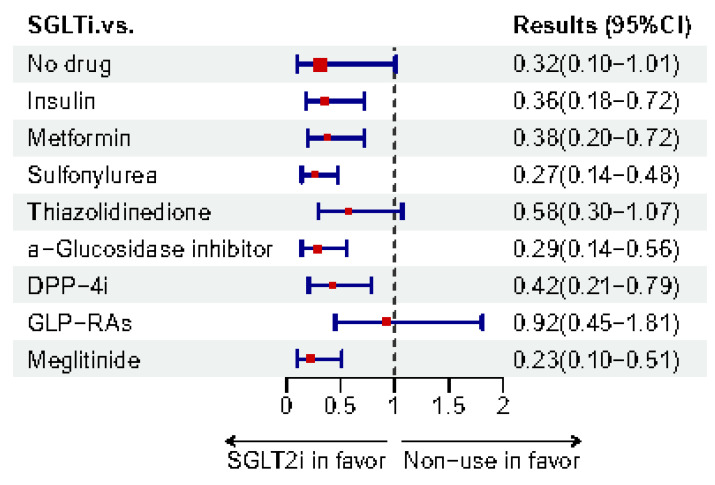
Network meta-analysis results of SGLT2i for lowering dementia risk compared to control or other interventions from Tian et al. [20].

**Figure 7:**
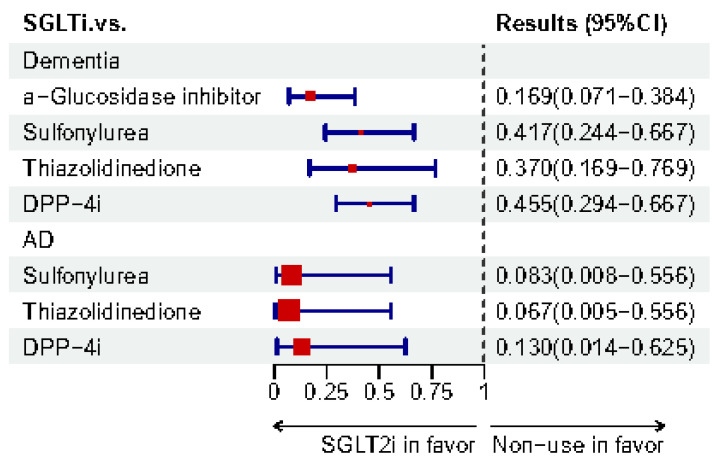
Bayesian network meta-analysis results of SGLT2i for lowering dementia and AD risk compared to control or other interventions from Sunwoo et al. [19].

Table 1 collects the clinical evidence of SGLT2i in treating CI in T2DM, while Figure 1, Figure 2, Figure 3 and Figure 4 (by R.4.4.1 of free, same for the Figure 5, Figure 6 and Figure 7) illustrate the results. In a 5-year cohort study of 106,903 individuals aged 66 years or older, SGLT2i was associated with a significantly lower risk of dementia (adjusted HR 0.80, 95%CI 0.71–0.89) compared to DPP-4i, with dapagliflozin showing the lowest risk (adjusted HR 0.67, 95%CI 0.53–0.0.84), followed by empagliflozin (adjusted HR 0.78, 95%CI 0.69–0.89) and canagliflozin with an insignificant risk (adjusted HR 0.96 95%CI 0.80–1.16) [116]. Another prospective cohort among frail elders over 65 years with T2DM and HFpEF found empagliflozin significantly associated with improved MoCA scores (OR 3.609, 95%CI 1.566–8.321, *p* = 0.003) [117]. In a Swedish longitudinal prospective cohort of 132,402 subjects, SGLT2i users who showed dementia at baseline had a significantly lower dementia mortality rate (adjusted HR 0.43, 95%CI 0.23–0.80) compared to non-users, while insulin (adjusted HR 1.34, 95%CI 1.23–1.45, *p* < 0.001) or SUs (adjusted HR 1.19, 95%CI 1.01–1.42, *p* < 0.05) increased the mortality risk [118]. A cross-sectional study in 2023 observed that SGLT2i had a protective effect on patients at risk of MCI (OR 0.337, 95%CI 0.135–0.843, *p* = 0.020) under multi-factor analysis by logistic regression [119]. Using multivariable regression analysis where the improvement in MoCA scores was the dependent variable, empagliflozin treatment was calculated to have a coefficient of 0.794 (*p* = 0.031) [120]. A cohort in Hong Kong, China, associated SGLT2i with a lower risk of PD in addition to dementia and cerebrovascular onset, compared to DPP-4i as shown by Cox regression [121]. A nested case-control in Denmark reported a significantly lower risk of dementia (multi-adjusted OR 0.58, 95%CI 0.42–0.81) with SGLT2i use [122]. Measuring cognition function by the RBANS, including immediate memory, delayed memory, viso-spatial/construction, language and attention, a secondary analysis of the SMART2D cohort reported that SGLT2i was associated with significantly increased language scores (adjusted coefficient 0.74, 95%CI 0.12–1.36, *p* = 0.019) among 138 patients with a mean age of 60.6 years in Singapore [123]. A later observational trial studying a new SGLT2i reported that henagliflozin might significantly improve the MoCA scores and plasma phosphorylated Tau protein among T2DM patients with MoCA lower than 26 [124].

A cohort study investigating cerebrovascular and cognitive benefits of SGLT2i as a treatment among patients with atrial fibrillation and T2DM reported that non-SGLT2i users had a significantly increased risk of ischemic stroke/TIA, ICH and incident dementia compared to SGLT2i treatment after 3-years of follow-up [125]. When reporting MASE as an outcome including stroke, myocardial infarction and all-cause death, a 1.1-year followed-up cohort showed a decreased risk (HR 0.81, 95%CI 0.70–0.94) when comparing empagliflozin initiation to DPP-4i [128]. In 2023, a population-based cohort study taking gender into account showed that men might obtain more benefits than women when using SGLT2i rather than GLP-1 RAs for the outcomes of MASEs (men/women, sub-distribution HR 0.78/0.99, 95%CI 0.66–0.93/0.77–1.28) and stroke (0.70/1.23, 0.42–1.16/0.59–2.56), which remains to be fully studied in the future [129]. Concentrating only on cardiovascular outcomes, adverse cardiovascular events, including fatal or nonfatal stroke and TIA in EMPA-REG OUTCOME, were determined to have insignificant risks: 1.18 and 0.85, respectively, when using empagliflozin compared to a placebo [130]. Study DECLARE TIMI 58 reported that dapagliflozin was insignificantly associated with ischemic stroke compared to the placebo, with a result of HR = 1.01 [131]. Also, studying the association between SGLT2i and cardiovascular outcomes, VERTIS revealed an insignificant link between ertugliflozin and fatal or nonfatal stroke [132].

Reporting both incident dementia and other subtypes as outcomes, a cohort concluded SGLT2i had a protective effect in reducing risk incidence for dementia (HR 0.705, 95%CI 0.506–0.984), AD (0.939, 0.721–1.222), VaD (0.44, 0.235–0.824) and other/mixed dementia (0.601, 0.296–1.220) [126]. Compared to sulfonylureas, SGLT2i, when newly used, were associated with a significantly lower risk of all-cause dementia, AD, VaD, expressed by a minus risk difference of −2.5% (95%CI −3.0% to −2.1%), −0.47% (−0.61% to −0.33%), −0.39% (−0.55% to −0.23%), respectively, with or without CKD and combined with other glucose-lowering drugs or not [141].

As for RCTs, one study including 55 patients in Nanjing, China, favored liraglutide over dapagliflozin for the enhancement of impaired olfactory neural activation and cognition capacity in T2DM [142]. Another RCT conducted in Italy reported that SGLT2i (canagliflozin, empagliflozin, dapagliflozin) did not improve the cognitive status significantly as tested by verbal fluency, the Babcock Story Recall Test and attentive matrices test, compared to either baseline or incretins [134]. In China, in 2022, one RCT, including 96 patients, reported dapagliflozin combined with CBT could significantly increase cognition function scores (all *p* < 0.001) of ADL (MD ± SD 6.18 ± 1.96), MMSE (2.59 ± 0.72), QOL-AD (10.69 ± 1.45) [135].

Here, six RCTs are summarized (one is not included in Table 1), comprising 14 cohorts, one cross-sectional and one case-control study. As VaD is rarely reported, the cerebral vascular outcomes in EMPA REG OUTCOME, DECLARE TIMI 58, VERTIS and MACE have been summarized as risk factors or accelerators of VaD to understand the effect of SGLT2i from another perspective. They all reported insignificant results.

RCTs of high quality exploring the association between SGLT2i and CI among T2DM patients are relatively rare, so the evidence is not firmly convincing. Given both the significant and insignificant results, some confounding factors should be taken into consideration. (1) First, the population recruited and region of conduct. The basic incidence of CI varies from ethnicity to ethnicity. Thus, the impact of whether one has been prescribed SGLT2i against T2DM progression or not would be different in different regions as a result of genes, environment, lifestyles or health service, etc. It is similar when it comes to gender, age and other personal characteristics for the same ethnicity. (2) Second, the number of participants in certain clinical trials. It is confusing as to whether significant or insignificant results from a trial with a limited number of subjects are representative or just random errors. The RCT with significant results by Ying Zhao et al. only recruited 96 actual subjects (Table 1) [135]. (3) Third, the intervention (exposure) and control (non-exposure). The specific SGLT2i used were different, and the control was either another antidiabetic agent or a placebo. Given the possible toxic effects of some antidiabetics, which can accelerate the onset of CI, the protective role of SGLT2i becomes doubtful when compared to a placebo or no drug use. The insignificant results from RCTs using a placebo as control here verify this concern. (4) Last but not least, the outcomes of interest. When using the incidence of CI related to T2DM as the outcome, the years of follow-up should be sufficiently long, or there might be bias. When using the cognition function scores to quantify the deterioration by T2DM, not only the amount of change but rate should be taken into consideration in the calculation for comprehensive exploration.

**Table 1:** Clinical trials studying the association between SGLT2i and CI and cerebrovascular risks.

Author	Design	Population (Region)	Intervention	Versus	Outcome		Effect Size	Results (95%CI)	*p* for Results
Ding et al. [119].	CS	222 middle-aged and elderly patients with T2DM (China)	SGLT2i	Non-use	MCI		OR	**0.337 (0.135–0.843)**	0.020
Wium-Andersen et al. [122].	N-CC	11,619 dementia cases and 46,476 control all with T2DM (Denmark)	SGLT2i	Non-use	Dementia		OR	**0.58 (0.42–0.81)**	
S. Low et al. [123].	C	138 patients with T2DM (Singapore)	SGLT2i	Non-use	RBANS		β	0.20 (−0.03–0.44)	0.091
					IM			0.38 (−0.04–0.79)	0.074
					DM			0.09 (−0.34–0.52)	0.677
					VS			−0.11 (−0.67–0.46)	0.703
					Language			**0.60 (0.10–1.11)**	0.019
					Attention			0.05 (−0.35–0.44)	0.806
Mone et al. [117].	C	162 Frail older adults > 65 years with T2DM and HFpEF (Italy)	Empagliflozin	Non-use	MoCA scores improvement		OR	**3.609 (1.566–8.321)**	0.003
P. Mone et al. [120].	C	166 frail elders with diabetes and CKD (USA)	Empagliflozin	Non-use	MoCA scores		MD	1.9 (1.04) †	
							β	0.794 (0.073–1.516)	0.031
Zhang et al. [124].	C	290 T2DM patients ≥ 45 years with MoCA ≤ 26 (henagliflozin, *n* = 135; non-use, *n* = 155) (China)	Henagliflozin	Non-use	MoCA scores improvement		OR	3.670 (2.224–6.056)	<0.0001
					Plasma p-Tau level improvement			2.595 (1.598–4.213)	<0.0001
Chen et al. [126].	C	2430 patients (age: 55–85 years) with AF and diabetes (Taiwan, China)	SGLT2i	Non-use	Dementia		HR	**0.705 (0.506–0.984)**	
					AD			0.939 (0.721–1.222)	
					VaD			**0.440 (0.235–0.824)**	
					Other/mixed			0.601 (0.296–1.220)	
					Stroke			**0.750 (0.601–0.937)**	
Riester et al. [133].	C	7710 elder aged ≥ 66 years with T2DM in nursing home (31.08% SGLT2i) (USA)	SGLT2i	GLP-1 RAs	MACE		HR	1.03 (0.74–1.44)	
A. Sharma et al. [129].	C	8026 patients (55.7% male) with T2DM at baseline (4231 SGLT2i users) (Australia)	SGLT2i	GLP-1 RAs	MACE	M	HR	**0.78 (0.66–0.93)**	
						F		0.99 (0.77–1.28)	
					Stroke	M		0.70 (0.42–1.16)	
						F		1.23 (0.59–2.56)	
Tang et al. [141].	C	35,458 new users aged ≥ 50 years of SGLT2i or SU with T2DM (USA)	SGLT2i	SU	Dementia		RD	**−2.5% (−3.0%, −2.1%)**	
					AD			**−0.47% (−0.61%, −0.33%)**	
					VaD			**−0.39% (−0.55%, −0.23%)**	
Mui et al. [121].	C	51,460 T2DM users with a median age of 66.3 years (Hong Kong, China)	SGLT2i	DPP-4i	Dementia		HR	**0.41 (0.27–0.61)**	<0.0001
					AD			0.25 (0.06–1.04)	0.0546
					PD			**0.28 (0.09–0.91)**	0.0349
					CVM			**0.36 (0.30–0.43)**	<0.0001
D. Edmonston et al. [128].	C	62,197 adult patients with T2DM (USA)	Empagliflozin	DPP-4i	MACE		HR	**0.81 (0.70–0.94)**	0.007
Riccardo Proietti et al. [125].	C	89,356 patients aged ≥ 18 years with AF and T2DM (global)	Non-use	SGLT2i	IS or TIA		HR	**1.12 (1.01–1.24)**	0.029
					ICH			**1.57 (1.25–1.99)**	0.001
					Dementia			**1.66 (1.30–2.12)**	0.001
Wu et al. [116].	C	106,903 Residents of Ontario, Canada ≥ 66 years old free of dementia (Canada)	SGLT2i	DPP-4i	Dementia		HR	**0.80 (0.71–0.89)**	
			Dapagliflozin					**0.67 (0.53–0.84)**	
			Empagliflozin					**0.78 (0.69–0.89)**	
			Canagliflozin					0.96 (0.80–1.16)	
J.Secnik et al. [118].	C	132,402 subjects with diabetes at baseline (Sweden)	SGLT2i	Non-use	Dementia death		HR	**0.43 (0.23–0.80)**	< 0.05
								0.52 (0.22–1.23) *	
Siao et al. [127].	C	976,972 patients with newly diagnosed T2DM (Taiwan, China)	SGLT2i	Non-use	Dementia		OR	**0.89 (0.82–0.96)**	0.0021
Perna et al. [134].	RCT	39 elder aged > 65 years subjects (women:16) with T2DM (Italy)	SGLT2i	Baseline	VFT		MC	0 (−4.25–4.25)	1
					BSRT			−1.32 (−3.36–0.71)	0.178
					AMT			−0.09 (−0.56–0.38)	0.676
Ying Zhao et al. [135].	RCT	96 patients with T2DM and MCI (China)	Dapagliflozin with CBT	Convention intervention	ADL		MD	**6.18 (1.96) †**	<0.001
					MMSE			**2.59 (0.72) †**	<0.001
					QoL-AD			**10.69 (1.45) †**	<0.001
EMPA REG OUTCOME	RCT	7028 adult patients with T2DM	Empagliflozin	Placebo	Stroke		HR	1.18 (0.89–1.56)	0.26
					TIA			0.85 (0.51–1.42)	0.54
VERTIS	RCT	8246 patients aged ≥ 40 years with T2DM	Ertugliflozin	Placebo	Stroke		HR	1.06 (0.82–1.37)	
DECLARE TIMI 58	RCT	17,160 patients aged ≥ 40 years with T2DM	Dapagliflozin	Placebo	IS		HR	1.01 (0.84–1.21)	

Non-use participants who did not use SGLT2i as a treatment. M, male; F, female. β, coefficient in multivariable regression analysis. Blank means not reported. Results in boldface mean significant effect size whose *p*-value < 0.05. * where the population were free of dementia at baseline and the OR stands for the mortality risk of dementia death. † the data here are mean and standard deviation: M(SD).

In the future, the effects of different SGLT2i choice and dosage could be tested, and the outcomes of interest in trials could include incidence of CI (MCI, dementia, AD, VaD), difference in cognition function scores (MMSE, MoCA, etc.) and deterioration rate of cognition among T2DM patients. More trials should concentrate on VaD in T2DM.

#### 3.1.2. Evidence-Based Meta-Analyses

Meta-analysis is considered as evidence of high quality. Therefore, meta-analyses regarding the use of SGLT2i to treat CI in T2DM were collected and are shown in Table 2. The forest plot (Figure 5) shows the results of different outcomes. Recently, in 2024, an umbrella systemic review and meta-analysis concluded that SGLT2i are associated with a reduced risk of dementia [15]. Compared with other antidiabetics, SGLT2i in a Bayesian network meta-analysis demonstrated a lower risk of dementia (vs. α-glucosidase inhibitors, RR 0.169, 95%CI 0.071–0.384; vs. DPP-4i, 0.455, 0.294–0.667; vs. SUs, 0.417, 0.244–0.667; vs. TZDs, 0.370, 0.169–0.769) and AD (vs. DPP-4i, 0.130, 0.014–0.625; vs. SUs, 0.083, 0.008–0.556; vs. TZDs, 0.067, 0.005–0.556) [19]. Meanwhile, another meta-analysis and network meta-analysis reported that SGLT2i might have the best potency for mitigating CI in T2DM. The meta-analysis used the methods of SUCRA (94%) [20]. Figure 6 and Figure 7 illustrate the risk-lowering effect of SGLT2i compared to control or other interventions. When cognition function was measured using the MMSE or MoCA, a meta-analysis that included one RCT and two cohorts showed that SGLT2i might contribute to a significant elevation (SMD 0.88, 95%CI 0.32–1.44) in cognition function scores [136]. In a meta-analysis in 2020 which included two case-control studies, SGLT2i showed an insignificantly decreased risk of dementia (OR 0.74, 95%CI 0.47–1.15) [137]. On the contrary, a new meta-analysis, including 12 RCTs, summarized that there is no significant effect on either diabetic dementia (OR 1.37, 95%CI 0.70–2.69) or subtypes of dementia (AD: 1.99, 0.59–6.71; PD: 0.63, 0.25–1.61; VaD: 0.40, 0.09–1.85) [138].

The results of Jaiswal et al. were calculated by a fourfold table, but others used logistic regression. That may be the reason why they raise a contrary conclusion. The reported heterogeneity was relatively large (e.g., 96.1%, 87%, 79%, 82.5%, 78.9%). According to funnel plots, the publication bias is acceptable.

Existing analyses have concluded that SGLT2i might be a promising choice in this situation. However, since there are fewer RCTs on this topic, the quality of meta-analysis remains low. An umbrella systemic review of high quality is needed in the future. Furthermore, more meta-analyses might provide evidence of optimal SGLT2i choice and dosage based on more clinical trials conducted in the future, especially different phases of RCTs.

**Table 2:** Results of meta-analysis reporting an association between SGLT2i treatment and CI in T2DM compared to non-SGLT2i.

Authors	Outcome	Effective Size	Results (95%CI)	I^2^ (%)	*p* for Heterogeneity	Studies Included (RCT/C/CS/CC)
Kuate Defo et al. [15].	Dementia	RR	**0.39 (0.20** **–** **0.76)**	96.1	0.000	5 (0/2/1/2)
Tian et al. [20].	CI	OR	**0.41 (0.22** **–** **0.76)**			
	Dementia		**0.41 (0.22** **–** **0.76)**			
	AD		**0.16 (0.07** **–** **0.39)**			
	CI	SUCRA	94.0%			12 (3RCTs/9OTs) *
M.Banerjee et al. [139].	Non-cardiovascular mortality	RR	**0.90 (0.82** **–** **0.99)**	0	0.75	8 (8/0/0/0)
Y.J. Youn et al. [136].	Dementia	HR	**0.68 (0.50** **–** **0.92)**	87	<0.001	4 (0/4/0/0)
		OR	0.74 (0.47–1.15)	79	0.03	2 (0/0/0/2)
	Dementia ≥ 60 years	HR	**0.84 (0.75** **–** **0.95)**			2 (0/2/0/0)
	Cognitive function scores	SMD	0.88 (0.32–1.44)			3 (1/2/0/0)
Tang et al. [140].	Dementia	RR	**0.62 (0.39** **–** **0.97)**	82.5	0.03	3 (0/1/0/2)
J.B. Zhou et al. [137].	Dementia	OR	0.74 (0.47–1.15)	78.9	0.03	2 (0/0/0/2)
Jaiswal et al. [138].	Dementia	OR	1.37 (0.70–2.69)	0		6 (6/0/0/0)
	AD		1.99 (0.59–6.71)	0		6 (6/0/0/0)
	PD		0.63 (0.25–1.61)	0		9 (9/0/0/0)
	VaD		0.40 (0.09–1.85)	0		4 (4/0/0/0)

Non-cardiovascular mortality includes infectious diseases, cancers, lung diseases, dementia, renal diseases and other external causes. Blank means not reported. Results in boldface mean significant effect size whose *p*-value < 0.05. * Three RCTs and nine observational trials were included.

### 3.2. Potential Mechanism SGLT2i as a Treatment

After reviewing the experiments and reviews, the neuroprotective effects against CI in T2DM could be simply divided into (1) a metabolism effect, (2) anti-inflammation and oxidative stress effects,(3) effects with neurotrophic factors, (4) an AChE inhibition effect. The metabolism effect is driven by the original function of anti-reabsorption to lower glycemia by inhibiting SGLT2 located in the kidney.

**Figure 8:**
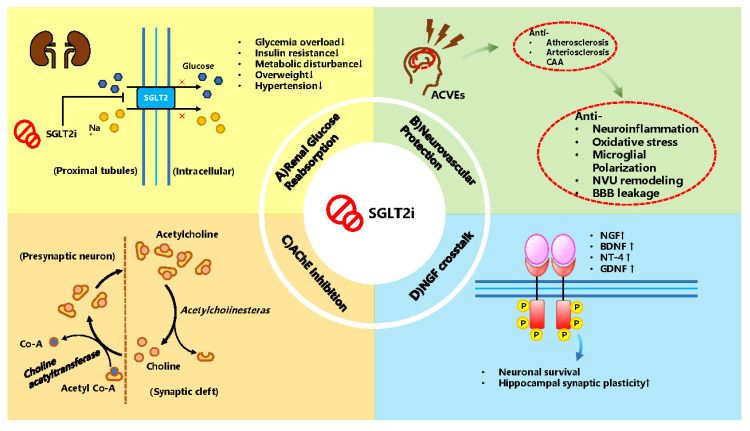
The hypothetical mechanism of the protective and treating effects when using SGLT2i. (**A**) Renal Glucose Reabsorption. SGLT2i inhibit renal glucose reabsorption to ameliorate glycemic overload, insulin resistance, metabolic disturbance, overweight and even hypertension, which is supposed to be the original power of neuroprotection. (**B**) Neurovascular protection. The metabolism benefits might manifest as anti-atherosclerosis, arteriosclerosis and CAA in the brain via anti-neuroinflammation, oxidative stress, microglial polarization, NVU remodeling and BBB leakage. (**C**) AChE inhibition and (**D**) NGF crosstalk. The possible extra effects of SGLT2i against CI by AChE inhibition, which is the therapeutic strategy of AD, and NGF crosstalk leading to neuronal survival and synaptic plasticity improvement in hippocampal. An upward arrow means increasing, improvement, or being up-regulated, which can be interpreted as any positive effect. A downward arrow indicates lightening, ameliorating or being down-regulated, which can be thought of as the antagonism of any disadvantage. Same for the Table 3 and Table 4.

**Figure 9:**
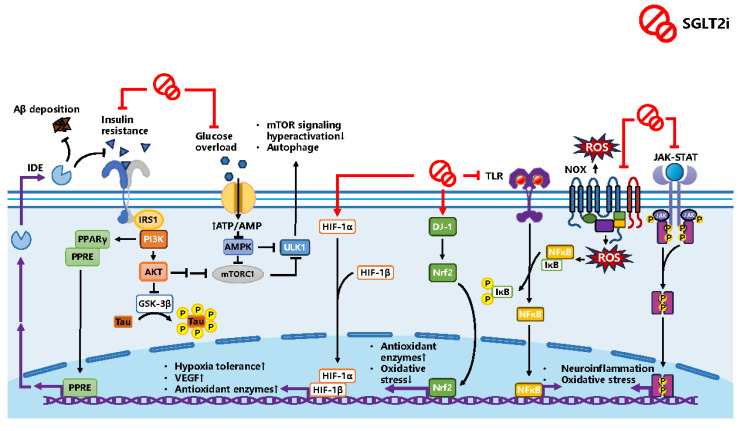
The hypothetical molecular mechanism and involved signaling pathways of SGLT2i against CI. The arrow indicates promotion, and the horizontal arrow indicates inhibition.

As a metabolic result, the body improves its metabolism homeostasis by modulating insulin and AMPK and mTOR signaling pathways, with blood pressure and body weight lowering, which have cardiovascular benefits. Not only does SGLT2i show anti-inflammatory and oxidative stress effects on nervous tissue but also on the vascular wall in the brain via regulating microglial polarization, cytokines, ROS and relative enzyme levels involved in NFκB, NLRP3 Inflammasome, HIF-1α and NADPH oxidase pathways. SGLT2i also crosstalks with neurotrophic factors, including BDNF, NT4 and GDNF, which have neuroprotective effects on neurons and neuroglia cells. Promisingly, experiments have shown that SGLT2i could act as dual inhibitors of SGLT2 and AChE, amplifying its treating potential in CI. Figure 8 and Figure 9, created by PowerPoint (office 2019), illustrate the hypothetical mechanism of the preventative and treating role of SGLT2i.

#### 3.2.1. Studied Mechanism in Animal Experiments 

In an SGLT2 spontaneous mutant mouse line (SAMP10-∆Sglt2), SGLT2 mRNA expression reduced to 34% compared to the SAMR (the senescence-resistant mouse model) 1 mice, and APP expression in the hippocampus showed significantly increased expression, while amyloid precursor-like protein (Aplp) was markedly downregulated, where the age-related decline in cognition was detected via membrane translocation of GLUT1 in the brain interfered with by APP and an unclear mechanism that increased APP expression associated with SGLT2 mutations [143]. In an experiment using a mice model of T2D-AD conducted in 2023, empagliflozin was reported to reduce p-Tau levels by activating ACE2/MasR that might crosstalk with IRS1/AKT/GSK3β pathways [86]. Table 3 summarizes some experiments investigating the potential mechanism using SGLT2i as a treatment of CI in T2DM. At the same time, another experiment reported empagliflozin and dapagliflozin as showing protective effects on diabetes-induced CI of diabetic mice, through modulating the expression of cytokines and proteins, including neurotrophic factors (BDNF, NT4), HIF-1α, APP and α-synuclein [144]. In 2021, empagliflozin was proven to attenuate CI in T2DM via suppressing inflammatory mediators, including IL-1β, IL-6 and TNF-α and oxidase stress in T2DM mice caused by a high-fructose diet [145]. In addition, empagliflozin also had a protective effect on NVU via ultrastructural remodeling discovered in type 2 diabetic db/db female mice by transmission electron microscopy [146]. Canagliflozin studied in male and female mice models also reported a neuroprotective effect against CI in aging by the reduction in the inflammatory response and mTOR signaling pathway in a sex-specific manner [147]. In addition to the mechanism mentioned above, a molecular structure experiment concluded that dapagliflozin had an inhibiting effect on AChE, which is the primary target of AD therapy [148]. Another experiment with a similar purpose reported that sotagliflozin and ertugliflozin might be dual inhibitors of SGLT2 and AChE [149].

**Table 3:** Animal experiments about SGLT2i in treating CI in T2DM. HFD, high-fat diet; STZ, streptozocin; HFruD, high-fructose diet; RVS, rivastigmine; US, ultrastructure; MasR, mitochondrial assembly receptor; NN, nitrite–nitrate; TBARS, thiobarbituric acid reactive substance; SOD, Superoxide dismutase; BM, base membrane.

Author	Model	Drugs	Control	Results	Mechanism
A.Y. Sim et al. [86].	Male C57BL/6 mice fed with HFD and injected STZ	Empagliflozin, 25 mg/kg/day, orally	Vehicle or DPP-4i	Hippocampal-dependent cognitive functions↑; Hepatic lipid accumulation↓ and inflammation↓; Promoting glucose homeostasis	Restore brain insulin signaling; pTau and Aβ↓: IRS1/AKT/GSK3β pathways
					pTau↓: ACE2/MasR↑
Iwona Piątkowska-Chmiel et al. [144].	CD-1 male mice with administration of 20% aqueous fructose solution and injection of freshly prepared STZ solution	Empagliflozin, 10 mg/kg/day	Vehicle	Protective effects of cognition	Inflammatory cytokines↓
					BDNF↑
					NT4↑
					α-synuclein in prefrontal cortex↑
		Dapagliflozin, 10 mg/kg/day	Vehicle	Protective effects of cognition	Inflammatory cytokines↓
					BDNF↑
					HIF-1α↑
					APP in hippocampus↑
T.Khan et al. [145].	CLB57/6 mice fed with HFruD	Empagliflozin, 4.4 mg/kg/day	Normal saline (10 mL/kg) or RVS (1.5 mg/kg)	Attenuating memory deficit	NN↓, TBARS↓, SOD↑, catalase↑
					IL-1β, IL-6, TNF-α↓
					TLR4-NFκB pathway↓
					GSK3β/pTau↓, Aβ (1–40)↓, Aβ (1–42)↓
M.R Hayden et al. [146].	Female db/db mice and wild control	Empagliflozin, 10 mg/kg/day		Ameliorating US remodeling of NVU	BM thickening↓, damage on BBB↓
					Activated microglia invasion↓
					Aberrant mitochondria remodeling↓
					myelin remodeling↓
					Axonal collapse↓
					Myelin disarray↓
Jayarathne et al. [147].	UM-HET3 mice	Canagliflozin, 14.4 mg/kg	Purina 5LG6	Insulin sensitivity↑; locomotor activity↑; overall behavior function↑	mTOR signaling↓
					Inflammatory response↓
					microgliosis↓, astrogliosis↓
Hierro-Bujalance et al. [150].	APP/PS1, db/db, APP/PS1 & db/db mice	Empagliflozin, 10 mg/kg		Learning and memory in AD, T2D and AD-T2D mice↑; Brain atrophy↓; Spontaneous bleeding↓	amyloid pathology↓
					Iba1^+^ burden↓
					tau phosphorylation↓

#### 3.2.2. Reviewed Mechanism

A review in 2023 summarized that SGLT2i might ameliorate CI via decreasing the leakage of the blood–brain barrier, reducing reactive oxygen species, Aβ levels and TNF-⍺, improving endothelial and microglia functions against neurodegenerative diseases such as AD and PD, although it was given limited space among other outcomes of interest [151]. Concentrating on LOAD, SGLT2i were concluded to be a novel pleiotropic antidiabetic drug class for LOAD and vascular brain damage for both direct (oxidative stress modulation; increase in brain-derived neurotrophic factor, BDNF; protective effect on neuronal loss and reduction in Aβ plaques; acetylcholinesterase, AChE, inhibition) and indirect (reducing blood pressure; improving cardiac function; modulating inflammation; promoting weight loss; reducing atherosclerotic plaque progression) neuroprotective effects compared to hypoglycemic agents and other anti-hyperglycemic agents [152]. Table 4 summarizes the reviewed mechanism of SGLT2i used to treat CI related to T2DM or diabetes. Serving as glucose sensors in response to extracellular glucose changes, SGLT2i showed their neuroprotective and neuro-cognitive effect in T2DM. However, the particular distribution and co-presence of SGLT1/SGLT2 in the CNS needs to be deeply studied as it might play a possible role in brain and cognitive activity [153]. The neuroprotective effects of SGLT2i have been reported to be related to atherosclerosis, inflammation, oxidative stress and mitochondrial dysfunction, mTOR signaling and cerebrovascular dysfunction, and differ in each classification of drug [154]. In a review mainly discussing the mTOR and AMPK/ULK1 pathways and metabolism homeostasis, it was reported that SGLT2i act as a metabolism regulator of glucose and amino acids, the sequence of which activated mTOR signaling calming and AMPK/ULK1 pathway acceleration to promote autophagy and mitophagy, which might be a beneficial target of AD treatment [155].

An article discussing the potential role of SGLT2i in treating cerebrovascular complications or accidents of diabetes suggested they could lower the risk of cerebrovascular dysfunction, including endothelial dysfunction, atherosclerosis, hypercoagulability, oxidative stress, renal reperfusion injury, which probably contribute to VaD [156]. When it comes to ischemia-related cerebral damage, which might contribute to VaD, empagliflozin, one of the SGLT2i, showed a neuroprotective effect at another stage against CI, related vascular disease in T2DM via increasing the expression of HIF-1α and VEGF-A, decreasing the expression of caspase-3, thus limiting the infarct volume [157].

A study to repurpose empagliflozin as a treatment for CI conducted among 21 participants aged ≥ 55 years without diabetes detected that the levels of phosphorylated IGF-1 receptor, phosphorylated insulin receptor, phosphorylated-in-tyrosine insulin receptor substrate-1 and phosphorylated AKT increased in neuronal derived extracellular vesicles after the administration of empagliflozin, suggesting that SGLT2i might be put into use in related disease via activating the IGF-1 and insulin/IR/IRS-1/PI3K/AKT pathways [158].

**Table 4:** Mechanism of SGLT2i treating CI in T2DM. Blank means not reported.

Authors	Outcomes	Potential Mechanism	
F. Mancinetti et al. [152].	LOAD and dementia	Direct effects	BP↓
			Cardiac function↑
			Weight↓
			Atherosclerotic plaque progression↓
		Indirect effects	Oxidative stress↓
			BDNF↑
			Aβ plaques↓
			AChE inhibition↑
M.R. Rizzo et al. [153].	Cognitive dysfucntion	Oxidative stress↓, NADPH oxidase↓	
		BDNF↑	
		Insulin sensitivity↑	
		Mitochondrial brain function↑	
		Synaptic plasticity↑	
		DJ-1/Nrf2 pathway	
		GDNF↑, PI3K/AKT/GSK3β (Ser9) pathway	
		NF-κB pathway↓, TNF-α levels↓	
		Brain extra- and intracellular accumulation of Aβ and NFTs↓	
		mTOR hyperactivation↓	
		AChE inhibition	
A. Pawlos et al. [154].	Neuroprotection	Canagliflozin	Anti-inflammatory
			Promoting M2 Macrophages Polarization
			Oxidative Stress↓
			mTOR Signaling↓
		Dapagliflozin	Anti-epileptic Potential
			CIMT Regression
			Anti-inflammatory
			NLRP3 Inflammasome↓
			Promoting M2 macrophage polarization
			Oxidative Stress↓
			mTOR Signaling↓
		Empagliflozin	BDNF↑
			CIMT Regression
			Anti-inflammatory
			Blood–Brain Barrier protection
			NLRP3 Inflammasome↓
			Promoting M2 macrophage polarization
			Oxidative Stress↓
			Neurovascular unit remodeling
			Cerebral ischemia/Reperfusion damage↓
			mTOR Signaling↓
		Ertugliflozin	Oxidative Stress↓
			mTOR Signaling↓
		Sotagliflozin	Oxidative Stress↓
S.V. Birajdar et al. [159].	Neurodegeneration	Insulin sensitivity↑	
		Oxidative Stress↓	
		Neuronal damage↓	
G. Goodarzi et al. [160].	AD	Empagliflozin	Oxidative Stress↓
			BDNF↑
			Infarct size
			HIF-1α/VEGF↑
		Canagliflozin	AChE inhibition
		Dapagliflozin	AChE inhibition
			Hippocampal synaptic plasticity↑
V. Chavda et al. [156].	CVAs	Endothelia dysfunction↓	
		glycemia↓	
		AGE superoxide↓	
		C-peptide and NFκB↓	
		Inflammation↓	
J Ariana Noel et al. [161].	CI	AChE inhibition	
		Oxidative Stress↓	
		Inflammation (NFkB)↓	
		mTOR pathway↓	
Mei et al. [162].	CI	NLRP3 inflammasome↓	
		ROS dependent neuronal apoptosis	
		Cerebral Glu metabolism↑	
		BDNF↑, NGF↑	
		AMPK pathway↑	
		CRP↓	
M.E Youssef et al. [163].	CI	Dapagliflozin	DJ-1/Nrf2 pathway
			GDNF↑
			PI3K/AKT/GSK3β pathway↑
			NFκB pathway↓, TNF↓
			AChE inhibition
		Empagliflozin	inflammatory mediators↓
			oxidative stress↓
			p-tau↓
			Aβ↓
Sim et al. [164].	AD	Peripheral insulin sensitivity↑	
		Body weight↓	
		Brain mitochondrial function↑	
		Insulin signaling↑	
		Cell death↓	
		Synaptic plasticity↑	
		mTOR inhibition	
		NLRP3 inflammasome-IL-1β modulation	
		Cerebral oxidative stress↓	
Riemma et al. [165].	CI	M2 microglial polarization↑	
		JAK2/STAT1 pathway↓	
		Free radicals↓, antioxidant enzymes↑	
	AD	mTOR activity↓	
		AChE inhibition, acetylcholine M1 receptor↑	
Michał Wiciński et al. [157].	Ischemia-related cerebral damage	Empagliflozin	HIF-1α↑
			VEGF-A↑
			caspase-3↓
			Limiting infarct volume
			Preventing US remodeling of NVU’s cell and myelin
Russell Esterline et al. [155].	AD	mTOR pathway↓	
		AMPK/ULK1 pathway↑	

## 4. Conclusions, Discussion and Perspectives

SGLT2i, not only in experiments but also in clinical trials, have shown their potential neuroprotective effects against damage from hyperglycemia and insulin resistance. To some extent, the results of the experiments and clinical trials have shown consistency, so the rationality of causal inference is probably convincing.

The outcomes of interest in clinical trials included the onset of CI and its subtypes and the decline in cognition function domains evaluated by rating scales, where both categorical variables (OR, RR, HR) and continuous variables (mean, MD, SMD) were used for description and meta-analysis. Insight from these clinical trials might be constructive starting points for investigations in the future. SGLT2i might have unique protective effects against VaD for verified cardiovascular benefits compared to other anti-hyperglycemia drugs. However, contrary results have been reported that cannot be overlooked. To conclude the convincing treatment effects of SGLT2i, comprehensive analysis and evaluation of those contradictory results are essential.

After summarizing the experiments and reviews, SGLT2i might prevent or alleviate CI among T2DM patients via ameliorating metabolism homeostasis, suppressing inflammation and oxidative stress, cooperating with neurotrophic factors and inhibiting AChE. Nevertheless, the deeper mechanism of SGLT2i and how they regulate the expression or activity of relevant molecules working in specific pathways could be explored in the future.

The promising role of SGLT2i as a treatment in CI in T2DM still needs to be deeply and fully studied and understood. Existing evidence is not enough to give a recommendation of specific SGLT2i as an optimal treatment. Clinical trials in this field, especially RCTs, should be conducted regarding SGLT2i in treating diabetic CI, to provide more evidence of its value in clinical practice. A meta-analysis and umbrella systemic review should also be used to provide evidence-based, convincing results on this topic in the future.

## Abbreviations

**Terms**	**Full Name**
DM	diabetes mellitus
T2DM	type 2 diabetes mellitus
IGT	impaired glucose tolerance
IFG	impaired fasting glucose
CI	cognitive impairment
MCI	mild cognitive impairment
AD	Alzheimer’s disease
VaD	vascular dementia
Aβ	amyloid β
NFTs	neurofibrillary tangles
CAA	cerebral amyloid angiopathy
OR	odds ratio
ADA	American Diabetes Association
MMSE	Mini-Mental State Examination
MoCA	Mini-Cog and Montreal Cognitive Assessment
SGLT2	sodium–glucose cotransporter 2
SGLT2i	sodium–glucose cotransporter 2 inhibitors
DPP-4i	dipeptidyl peptidase-4 inhibitors
SUs	sulfonylureas
TZDs	thiazolidinediones
GLP-1 RAs	glucagon-like peptide 1 receptor agonists
SUCRA	surface under the cumulative ranking curves
ACVEs	adverse cerebral vascular events
GSK3β	glucogen synthase kinase 3β
IR	insulin receptor
IRS	insulin receptor substrate
PI3K	phosphoinositide 3-kinase
AKT	protein kinase B (PKB)
BBB	blood–brain barrier
CNS	central neural system
IDE	insulin-degrading enzyme
APP	amyloid-β precursor protein
PPAR	peroxisome proliferators-activated receptors
NFκB	nuclear factor κ B
NO	nitric oxide
IL	interleukin
TNF	tumor necrosis factor
ROS	reactive oxygen species
NADPH	nicotinamide adenine dinucleotide phosphate
NOX2	NADPH oxidase 2
AGEs	advanced glycation end-products
HR	hazard ratio
HbA1c	glycosylated hemoglobin A1c
CRP	C-reactive protein
CKD	chronic kidney disease
HRQoL	health-related quality of life
RCTs	randomized control trials
HFpEF	heart failure with a preserved ejection fraction
95%CI	95% confidence interval
RBANS	Repeatable Battery for the Assessment of Neuropsychological Status
IS	ischemic stroke
TIA	transient ischemic attack
ICH	intracranial hemorrhage
MACEs	major adverse cardiovascular events
CBT	cognitive behavioral training
ADL	activity of daily living
MD	mean difference
SD	standard deviation
QOL-AD	the quality of life AD scores
C	cohort
CS	cross-sectional
N-CC	nested case-control
PD	Parkinson’s disease
CVM	cerebrovascular mortality
VFT	verbal fluency test
BRST	Babcock story recall test
AMT	attentive matrices test
IM	immediate memory
DM	delayed memory
VS	visuospatial/construction
MC	mean changes
RD	risk difference
SMD	standard mean difference
RR	risk ratio
AChE	acetylcholinesterase
AMPK	AMP-activated protein kinase
mTOR	mammalian target of rapamycin
HIF	hypoxia-inducible factor
NLRP3	nucleotide-binding oligomerization domain, leucine-rich repeat and pyrin domain-containing 3
ACVEs	adverse cerebral vascular events
NVU	neurovascular unit
NGF	nerve growth factor
BDNF	brain-derived neurotrophic factor
NT-4	neurotrophins 4
GDNF	glial cell-derived neurotrophic factor
PPRE	peroxisome proliferators reactive element
Nrf2	nuclear factor-erythroid 2-related factor 2
TLR	toll-like receptor
IκB	inhibitor of NFκB
JAK-STAT	Janus Kinase-signal transducer and activator of transcription
ACE2	angiotensin-converting enzyme 2
LOAD	Late-onset AD
VEGF-A	vascular endothelial growth factor A
IGF-1	insulin-like growth factor-1
